# The role of 5-HT1A receptors in mediating acute negative effects of antidepressants: implications in pediatric depression

**DOI:** 10.1038/tp.2015.57

**Published:** 2015-05-05

**Authors:** K A Rahn, Y-J Cao, C W Hendrix, A I Kaplin

**Affiliations:** 1 Department of Psychiatry and Behavioral Sciences, Johns Hopkins University, Baltimore, MD, USA; 2 Department of Neurology, Johns Hopkins University, Baltimore, MD, USA; 3 Division of Clinical Pharmacology, Department of Medicine, Johns Hopkins University, Baltimore, MD, USA

## Abstract

Acute antidepressant exposure elevates the frequency of impulsive behavior and suicidal thoughts in children and adolescents with major depressive disorder (MDD). Long-term antidepressant treatment, however, is beneficial for pediatric MDD, so it is necessary to explore novel treatments that prevent the potentially dangerous consequences of acute antidepressant initiation. In the present study, a treatment strategy designed to reverse the acute negative behavioral effects of antidepressants was tested in rodents. Co-administration of the 5-HT1A receptor (5-HT1AR) antagonist WAY-100635 reversed the negative effects of acute fluoxetine, a serotonin reuptake inhibitor, but not reboxetine, a norepinephrine reuptake inhibitor, supporting the involvement of 5-HT1AR in mediating the negative consequences of acute selective serotonin reuptake inhibitor (SSRI) treatment. No 5-HT1AR antagonists are currently approved for use in pediatric populations, so alternative strategies should be explored. One such strategy was suggested based on the hypothesis that the rate of 5-HT1AR activation and the subsequent inhibition of serotonergic neuron activity caused by acute SSRI administration is proportional to the loading rate of an antidepressant. Existing pharmacological data were examined, and significant correlations were observed between the half-life of antidepressants and the rate of suicide-related events (SREs). Specifically, antidepressants with longer half-lives have lower rates of SREs. On the basis of these data, novel dosing strategies were developed for five antidepressants to mimic the pharmacological profile of the antidepressant with the longest half-life, fluoxetine. These dosing strategies could be used to decrease the rate of SREs associated with acute antidepressant treatment in pediatric MDD until an improved pharmacological treatment is developed.

## Introduction

Major depressive disorder (MDD) occurs in ~8% of all children and adolescents^1^ and can negatively impact social, cognitive and emotional development. Suicide is the third leading cause of death in adolescents and young adults aged 10–24 years^2^ and the second leading cause of death in young adults 25–34 years.^3^ Although psychotherapeutic interventions such as cognitive behavioral therapy may be sufficient to treat those with mild or moderate depression, successful treatment of MDD in children and adolescents often requires the addition of pharmacological intervention. The Food and Drug Administration (FDA) has approved only one antidepressant for the treatment of MDD in children and adolescents 8–18 years, the selective serotonin reuptake inhibitor (SSRI) fluoxetine. Another SSRI, escitalopram, has FDA approval for the treatment of MDD in adolescents of 12–17 years.

In October 2004, the FDA conducted a meta-analysis of 24 double-blind placebo-controlled trials of pediatric SSRI treatment and found an increased risk of suicidal thoughts and behaviors in children and adolescents initiating SSRI treatment (four, versus 2% in placebo controls).^4, 5, 6^ On the basis of these findings, the FDA issued a black box warning and guidelines that include the requirement for weekly examinations to monitor suicidal thoughts and behaviors during the first month of pediatric antidepressant treatment. This warning was extended to include young adults (18–24 years) in 2007.^7^ More recent behavioral data collected from 15–99-year-old subjects treated with SSRIs revealed a 2.6% increase in the frequency of suicidal ideation and a 4.6% increase in the frequency of preparatory actions for suicide per year-of-age decrease of the subject,^8^ further supporting the concept that acute SSRI-mediated suicidal ideation is particularly relevant in pediatric populations. Although these data underscore the potentially risky early side effects of antidepressants, a host of epidemiological data support the long-term use of SSRIs for the treatment of depressed adolescents and children, both alone^1, 9^ and in combination with cognitive behavioral therapy.^10^

SSRIs prevent the reuptake of serotonin (5-HT) by blocking presynaptic 5-HT transporters, thereby increasing the availability of synaptic 5-HT to stimulate the postsynaptic neuron and, through additional mechanisms still not completely understood, lead to the desired downstream antidepressant effect.^11^ There is, however, up to a 2–4-week delay in the clinical onset of antidepressant effects, during which time the excess synaptic 5-HT activates 5-HT1A autoreceptors (5-HT1ARs) on the presynaptic neuron to inhibit further release of 5-HT.^12^ The desensitization of 5-HT1AR and recovery of proper serotonergic neuron firing occurs after ~2–4 weeks of exposure to SSRIs, which correlates well with the observed delayed onset of SSRIs efficacy.^13^

How might acute SSRI treatment increase suicidal ideation in children and adolescents? Pooled analyses from double-blind placebo-controlled studies reveal that acute antidepressant treatment increases suicidal thinking and behavior in pediatric populations,^5^ but a closer examination of the analysis of pooled pediatric study data reveals a relative risk of 0.93 for the emergence of suicidal thoughts and behaviors due to antidepressant treatment.^6^ This observation suggests that acute antidepressant treatment does not create suicidal ideation where there was previously none, but that acute antidepressant treatment makes children and adolescents more likely to act on pre-existing suicidal thinking. This increased incidence of actionable suicidal behavior could be defined as increased impulsivity or aggressive behavior as a result of acute SSRI treatment. In line with this hypothesis, acute SSRI administration causes a well-documented reduction in serotonergic neuron firing,^14^ and numerous clinical studies report that hyposerotonergic states (that is, low cerebrospinal fluid levels of the 5-HT metabolite 5-HIAA) correlate with increased impulsive and violent behavior.^15, 16^ Because hyposerotonergic states are associated with increased impulsivity and aggressive behavior, and because pediatric populations have increased rates of suicidal ideation and impulsivity during the first month of SSRI treatment,^17, 18^ it is plausible that the acute negative effects of SSRIs could be a result of the 5-HT1AR-mediated decrease in serotonergic output during acute treatment.

Acute SSRI exposure activates 5-HT1AR, which decreases serotonergic neuron output. The rate at which SSRIs acutely stimulate 5-HT1AR is expected to be proportional to the maximum concentration of the drug following dosing (*C*_max_). Thus, the negative behavioral effects caused by acute SSRI treatment should be proportional to the rate at which SSRI concentration rises in the brain. Functional half-life (*t*_1/2_) is inversely proportional to the natural logarithm of *C*_max_/*C*_min,_^19^ and this relationship can be used to test the prediction that rapid SSRI loading and clearance rates account for the suicidal ideation seen in pediatric populations. More specifically, if the 5-HT1AR-mediated decrease in serotonergic output accounts for the increased suicidal ideation, then antidepressants that lead to a more moderate activation of 5-HT1ARs should have a lower rate of suicidal ideation in children and adolescents. SSRI-mediated suicide-related events (SREs) have been reported in a meta-analysis of double-blind placebo-controlled studies.^20^

There is a pressing need for enhancing and improving treatment strategies in pediatric MDD, as recent declines in antidepressant use have coincided with increased suicidal behavior in young adults.^21^ The first goal of this project is to preclinically demonstrate that the negative effects of acute SSRI exposure is mediated by 5-HT1AR activation and can be blocked with a 5-HT1AR antagonist. The negative acute effects of SSRIs can be modeled in rodent studies, where acute SSRI exposure causes anxiety and fear, whereas chronic SSRI exposure decreases anxiety and fear.^22, 23, 24^ If the 5-HT1AR-mediated decrease in serotonergic output accounts for the increased suicidal ideation in children and adolescents, then antidepressants that lead to a less robust activation of 5-HT1ARs (that is, a slower loading rate) should cause lower levels of suicidal ideation and behavior in pediatric populations. Therefore, as a further test of the hypothesis that 5-HT1AR activation leads to a hyposertonergic state associated with increased SREs, the second goal of this project was to demonstrate a relationship between the rates at which SSRI concentrations rise in the brain and the rate of SREs in a treatment-matched pediatric population. Fluoxetine is the SSRI that is least likely to increase the relative risk of suicidal ideation in children and adolescents,^20^ consistent with its slow initial loading rate that produces a relatively small hyposeratonergic state through the modest stimulation of 5-HT1AR. On the basis of this knowledge, the final goal of the project was to use a computer-generated dosing simulation to develop novel treatment regimens for existing SSRIs based on the pharmacological properties of fluoxetine, the SSRI with low 5-HT1AR activation that is least likely to increase the relative risk of suicidality in children and adolescents.^20^ These novel dosing strategies would therefore be expected to maximize the safe administration of other SSRIs, approaching that observed with fluoxetine, while minimizing their negative effects (that is, SREs).

## Materials and methods

### Animals and housing

Eight-week-old male C57BL/6 mice were obtained from the National Cancer Institute (Frederick, MD, USA) and housed in the Broadway Research Building Johns Hopkins animal facility. Housing and behavioral testing rooms were temperature controlled with a 12-h light/dark cycle, and all behavioral tests were conducted during the light cycle. Animal protocols were approved by the Johns Hopkins University School of Medicine Animal Care and Use Committee.

### Drug administration

For 7 days prior to behavioral testing, vehicle (phosphate-buffered saline) was delivered to each mouse via a 0.1-ml intraperitoneal injection. On testing days, phosphate-buffered saline, fluoxetine (20 μg ml^−1^, Sigma-Aldrich, St Louis, MO, USA), WAY-100635 (0.3 mg kg^−1^, Sigma-Aldrich) and/or reboxetine (5 mg kg^−1^, Sigma-Aldrich) were delivered 40 min prior to the commencement of testing.

### Elevated plus maze

Mice (*n*=10) were tested on the elevated plus maze in an isolated behavior suite. Mice were placed in the center of the maze and given 5 min to freely explore the maze. Mice were tracked using ANY-maze software (Stoelting, Wood Dale, IL, USA).

### SSRI clearance vs SREs

The odds ratio of SREs in pediatric populations due to SSRI exposure was reported by Smith in 2009 (ref. 25) based on a 2004 FDA report.^20^ The natural log-transformed half-life (ln(*t*_1/2_)) of three SSRIs in pediatric populations was calculated from the published pediatric *t*_1/2_ of citalopram,^26^ sertraline,^27^ paroxetine.^28^ The *t*_1/2_ of fluoxetine is equal in children, adolescents and adults,^29^ so the *t*_1/2_ reported in adults was utilized in the present study. Blood clearance rates of six SSRIs in adults were calculated from the published adult *t*_1/2_ of fluoxetine, citalopram, sertraline, paroxetine, venlafaxine and fluvoxamine.^25^ The ln(*t*_1/2_) of the SSRIs was plotted against the odds ratio of SREs associated with each individual SSRI for children and adults.

### Antidepressant dosing simulation

The time course of the fluoxetine concentration in blood plasma was simulated based on the following assumptions: (1) the pharmacokinetics (PKs) of fluoxetine can be described with a model of first-order absorption and Michaelis–Menten elimination: d*C*/d*t*=K01 × (D/V) × exp (−K01 × *t*)−*V*_m_ × *C*/(*K*_m_+*C*), where *C* is the concentration at time *t* after a single dose *D*; *V*, K01, *V*_m_ and *K*_m_ are apparent volume of distribution, first-order absorption constant, maximum elimination constant and Michaelis constant, respectively; (2) the body weigh was 70 kg and Prozac was administered as 40 mg once daily; and (3) peak drug concentration (*C*_max_), time to reach *C*_max_ (*T*_max_) and half-life (*t*_1/2_) of fluoxetine following a single 40-mg dose of Prozac were 35 ng ml^−1^, 7 h and 48 h, respectively.^30^ The final simulated model parameters were *V*, 1033 l; K01, 0.53 h^−1^; *V*_m_, 6.5 ng ml^−1^ h and *K*_m_, 448 ng ml^−1^; the simulated steady-state mean concentration of 149 ng ml^−1^ was apparently reached by day 25. The daily mean concentrations of fluoxetine as a percentage of the steady-state mean concentration were set as the target of daily mean concentrations for dosing paroxetine, citalopram, sertraline, venlafaxine and fluvoxamine. To simulate the dosing regimen, a one-compartment PK model with first-order absorption and elimination was used for sertraline,^31^ citalopram^32^ and venlafaxine with its active metabolite, O-desmethylvenlafaxine. *V*, *t*_1/2_ and *T*_max_ were assumed to be 1400 l, 26 h and 6.5 h for sertraline,^33^ 840 l, 35 and 4 h for citalopram^34^ and 426 l, 16.9 h^35^ and 8 h^35^ for venlafaxine, respectively. As the metabolic pathway of paroxetine^36^ and fluvoxamine^33^ at the therapeutic dose may be saturated, the Michaelis–Menten kinetic model was used to simulate their dosing regimen. *C*_max_, *T*_max_ and *t*_1/2_ were assumed to be 17.6 ng ml^−1^,^37^ 6.3 h^37^ and 16 h^38^ for paroxetine after a single 30-mg dose, and 30 ng ml^−1^, 6 h and 19 h^39^ for fluvoxamine after a single 50-mg dose, respectively. MATLAB Student Version Release 14 with Service Pack 1 (The MathWorks, Natick, MA, USA) was used to find out Michaelis–Menten kinetic parameters and all dosing regimens. Phoenix WinNonlin 6.3 (Certara, St Louis, MO, USA) was used to generate the time course of drug concentrations for a given dosing regimen.

### Statistical analyses

Statistical analyses were completed using GraphPad Prism 5.0 (La Jolla, CA, USA). One-way analysis of variance was used for comparisons between groups in the animal studies, and Pearson correlation analyses were used for SRE vs *t*_1/2_ or *C*_max_ data. *P*-values <0.05 were considered statistically significant. The Johns Hopkins Institute for Clinical and Translational Research Biostatistics Center provided statistical support.

## Results

**Figure 1:**
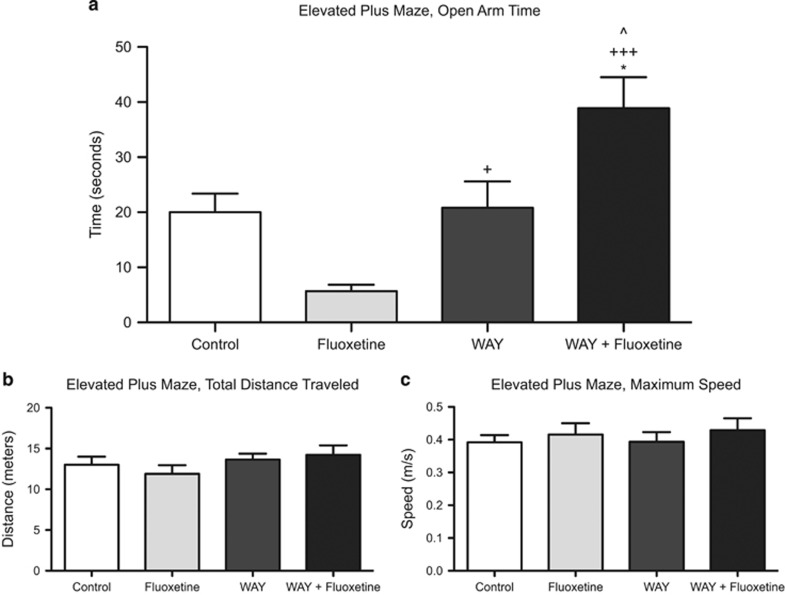
Mice treated with fluoxetine are more anxious in an elevated plus maze compared with control mice, but treatment with the 5-HT1AR antagonist WAY has no effect (**a**). Co-administration of WAY with fluoxetine reverses the anxiogenic effects of fluoxetine treatment alone and increases anxiolytic effects compared with control and WAY mice. No differences in total distance traveled (**b**) and maximum speed (**c**) in the maze were observed between groups, indicating that drug treatment did not affect activity and mobility, respectively. Significantly different from control at *P<0.05; significantly different from fluoxetine at ^+^*P*<0.05, ^+++^*P*<0.001; significantly different from WAY at ^*P*<0.05. *n*=8–10 mice per group.

Chronic administration of SSRIs has an anxiolytic effect on mouse behavior, whereas acute administration of SSRIs is anxiogenic.^23, 24^ Present studies confirmed the anxiogenic effects of acute fluoxetine administration, as assessed by time spent in open arms of the elevated plus maze. Mice given one dose of fluoxetine (20 mg kg^−1^) spent 72% less time in the open arms compared with control mice (5.67±3.68 vs 20.03±10.12 s, respectively) (Figure 1). Administration of the potent and selective 5-HT1AR antagonist WAY-100635 (0.3 mg kg^−1^) had no effect on time spent in the open arms compared with control mice (20.82±15.18 vs 20.03±10.12 s). Co-administration of WAY-100635 with fluoxetine, however, reversed the acute anxiogenic effects of acute fluoxetine, and WAY+fluoxetine-treated mice spent significantly more time exploring the open arms of the elevated plus maze compared with mice treated with fluoxetine alone (38.9±15.94 vs 5.67±3.68 s, *P*<0.001). Total distance traveled in the elevated plus maze and maximum speed did not differ between groups (Figure 1b and c), indicating that the fluoxetine and WAY treatments selectively affected anxiety but neither activity nor mobility. To confirm that the ability of WAY-100635 to block the anxiogenic effect of acute antidepressant treatment was specific to co-administration with SSRIs and not norepinephrine reuptake inhibitors, the acute effects of reboxetine and WAY-100635+reboxetine treatments were assessed. Mice given WAY-100635+reboxetine spent equal time in the open arms of the maze as compared with mice given reboxetine alone (13.79±11.3 vs 10.6±10.8 s).

**Figure 2:**
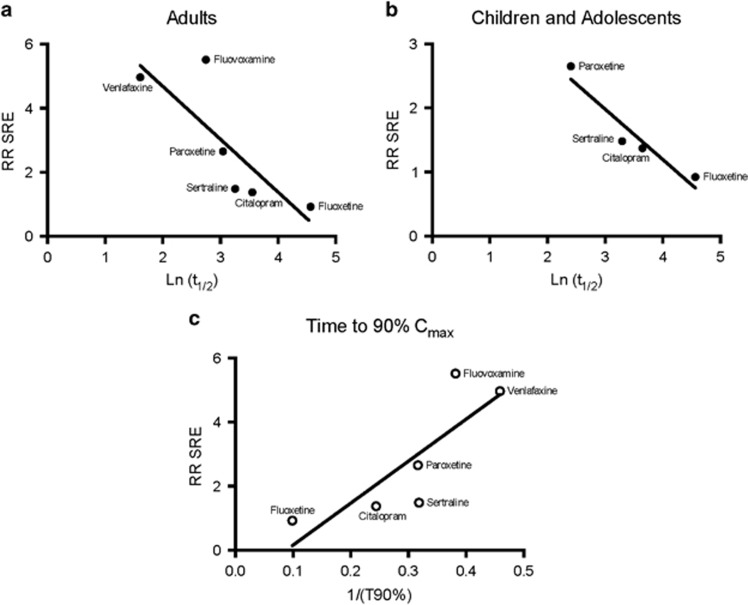
There is a significant relationship between the log-transformed *t*_1/2_ (Ln (*t*_1/2_)) of SSRIs vs the RR SREs for the given SSRI in adults (**a**) and children and adolescents (**b**). A thorough literature search uncovered *t*_1/2_ data of six SSRIs for adult analysis (fluoxetine, citalopram, venlafaxine, sertraline, paroxetine and fluvoxamine) and four SSRIs for pediatric analysis (fluoxetine, citalopram, sertraline and paroxetine). A significant positive correlation was observed between the two factors in both populations (*P*<0.05). There is also a significant relationship between the inverse of the time to 90% (1/(*T*90%)) of maximum concentration (*C*_max_) of that drug when it is administered on a constant dosing schedule and the risk of SREs (**c**, *P*<0.05). RR SRE, relative risk of suicide-related event; SSRI, selective serotonin reuptake inhibitor.

The risk ratios of SREs in pediatric patients for six antidepressants (fluoxetine, citalopram, venlafaxine, sertraline, paroxetine and fluvoxamine) were obtained from a report by the FDA that included 24 placebo-controlled clinical trials conducted in nine drug development programs.^6^ A thorough literature search uncovered *t*_1/2_ data for all six of these SSRIs only for adults, whereas the *t*_1/2_ data for only four SSRIs (fluoxetine, citalopram, sertraline and paroxetine) have been reported in pediatric populations. Thus, for completeness, we first plotted the natural log of *t*_1/2_ using the available values published for adults for all six SSRIs versus the rate of SREs in children and adolescents prescribed by the corresponding SSRI (Figure 2a). The natural log of the *t*_1/2_ was also plotted for the four SSRIs for which *t*_1/2_ data are available versus SREs in pediatric populations prescribed by the corresponding SSRI (Figure 2b). A significant positive correlation was found between the two factors in both populations, indicating that the faster the loading rate of a SSRI, the higher the chances of an SRE occurring (*P*<0.05). Furthermore, a significant relationship was observed between the inverse of the calculated time to 90% of maximum drug concentration (*C*_max_) during a constant dosing schedule and the rate of SREs for the given SSRI (*P*<0.05, Figure 2c).

**Figure 3:**
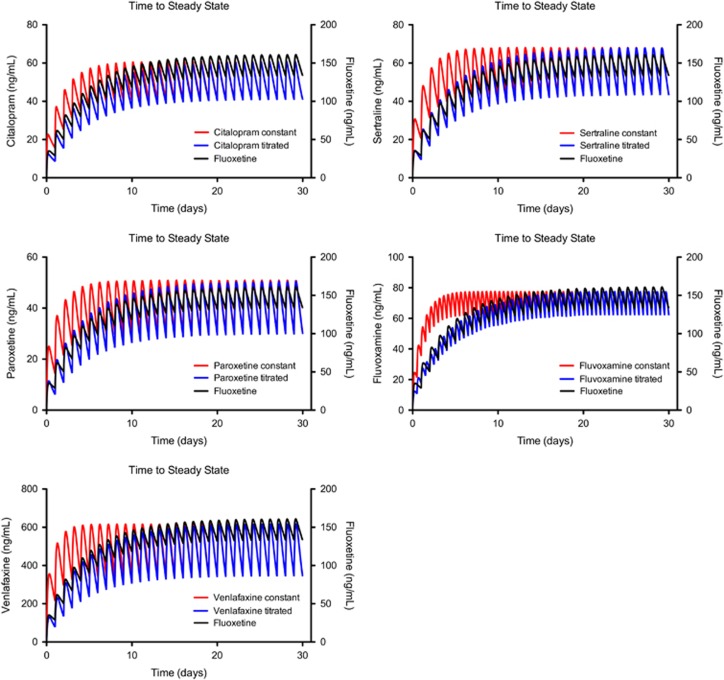
Simulated time course of blood concentrations for paroxetine, citalopram, sertraline, fluvoxamine and venlafaxine based on a constant dosing regimen (that is, how the drugs are currently prescribed according to the manufacturer's instructions) and a titrated dosing regimen to closely match the pharmacokinetics of fluoxetine. The concentration time course for fluoxetine was generated according to the manufacturer's instructions.

Fluoxetine, the antidepressant with the longest half-life and correspondingly slowest loading rate, is the SSRI with the lowest relative risk of SREs (0.92) in pediatric populations.^20^ Although it is not possible to change the PK properties of the other SSRIs to approximate those of fluoxetine, an alternative is to try to use a dose-loading strategy to make the rate at which other SSRIs load into the body approximate as closely as possible that of fluoxetine. A dosing curve of fluoxetine following the standard dosing regimen of 40 mg per day was generated using the computer program MATLAB. Drug concentrations approached steady state after 25 days. On the basis of the generated fluoxetine curve, simulated dosing regimens were calculated and generated by MATLAB for paroxetine, citalopram, sertraline, venlafaxine and fluvoxamine to make the trough blood concentration of each of these drugs approximate that seen with fluoxetine (Figure 3). These simulated regimens have been converted into a table (Table 1) to serve as dosing recommendations for the first month of SSRI treatment.

## Discussion

Treatment of MDD with an SSRI causes a small but significant elevated risk of suicidal behavior in children and adolescents^5, 6^ The present study was designed to better understand the mechanisms by which acute SSRI administration in children and adolescents results in increased SREs, and from this knowledge develop novel treatment strategies to combat this elevated risk of suicidal thoughts and actions. Activation of 5-HT1AR in the dorsal raphe nucleus results in autoregulation with a block of central nervous system sertonergic neuron firing, effectively leading to a relatively hyposerotonergic state, which has previously been shown to correlate with impulsive and aggressive behavior as seen in pediatric populations during acute SSRI dosing. We hypothesized that blockade of 5-HT1AR would lead to a decrease in the acute negative effects of SSRI treatment, which has implications for the increased rates of suicidal ideation in the initial weeks of antidepressant treatment through inhibition of the feedback loop and prevention of decreased serotonergic output. The combination drug treatment strategy of a 5-HT1AR antagonist administered in combination with a SSRI reversed the acute anxiogenic effects of a SSRI alone in rodents. We are hopeful that this treatment strategy can be developed and tested for future use in humans. Because no 5-HT1AR antagonists are currently approved for use in the clinic for this indication, however, we conducted analyses, based on our preclinical findings of the role of 5-HT1AR activation in mediating the acute negative effects of SSRI, demonstrating that pharmacological properties of SSRIs are related to the rates of SREs in children and adolescents. Previous studies have reported a link between antidepressant half-life and the incidence of SREs;^25^ however, the present study provides evidence for a mechanism of action to account for this association and is the first to include pediatric pharmacological data. On the basis of these analyses, we modified the dosing of existing drug treatments for MDD to mimic fluoxetine and minimize the risk of SREs associated with the first few weeks of antidepressant treatment. Until a pharmacological treatment can be developed for use in pediatric populations to decrease the acute risk of suicidal ideation and self-harm, these novel dosing strategies could allow for safer antidepressant treatment in pediatric MDD.

Pharmacological antidepressant treatment can be evaluated preclinically in rodents. Mice with a genetic disruption in 5-HT neurotransmission display depressive behaviors, heightened anxiety to conditioned stimuli and decreased anxiety to novel objects (that is, impulsivity).^40^ Similar to humans, mice exhibit anxious behaviors following acute antidepressant treatment that disappear following chronic exposure to a SSRI.^41^ In the current project, we have demonstrated a method of blocking and reversing the negative acute SSRI-mediated anxiogenic behavior in mice using a 5-HT1AR antagonist. Because our results show that WAY-100635+fluoxetine combination treatment causes increased anxiolytic behavior in mice as compared with either controls or mice treated with WAY-100635 alone, we speculate that the WAY-100635 and fluoxetine are working synergistically to increase 5-HT signaling. Future studies are required to confirm this effect. Co-administration of a 5-HT1AR antagonist with a SSRI doubles the synaptic 5-HT concentration after days compared with administration of a SSRI alone.^42^ Therefore, we predict that the anxiolytic effects in mice of SSRI treatment in conjunction with 5-HT1AR antagonist, which facilitates rapid and robust upregulation in synaptic 5-HT levels and serotonergic neuron activity, may have a similar effect in humans and improve mood and inhibit impulsivity and self-harm behaviors that are common during the first few weeks of antidepressant dosing in children and adolescents.

The importance of regulating 5-HT1AR activation levels during acute antidepressant treatment is supported by multiple independent studies. Genetic manipulation of presynaptic 5-HT1AR density in mice converted the rodents from SSRI treatment nonresponders (that is, high 5-HT1AR density and no benefit from SSRI treatment) to SSRI treatment responders (that is low 5-HT1AR density and benefit from SSRI treatment).^43^ Knockdown and complete knockout of 5-HT1AR in mice elevated the release of 5-HT in response to stressful stimuli, indicating that 5-HT1AR-mediated feedback inhibition powerfully regulates serotonergic neurotransmission.^44^ A positron emission tomography study reported that antidepressant-naive patients with MDD have a higher 5-HT1AR density compared with healthy controls and antidepressant-exposed patients with MDD.^45^ In addition, 5-HT1AR density was found to be significantly upregulated in the midbrain of suicide victims with MDD compared with normal non-depressed controls.^46^ A positron emission tomography and electroconvulsive therapy study recently reported a significant decrease in the binding of WAY-100635, the 5-HT1AR antagonist used in the present study, in patients successfully treated for MDD with electroconvulsive therapy,^47^ demonstrating that 5-HT1AR density decreases as symptoms of depression are alleviated. Taken together, these studies not only highlight the role of 5-HT1AR in the manifestation and/or treatment of depression, but also suggest that the receptor has equivalent roles in rodents and humans. Previous clinical studies have employed the co-administration of an SSRI with pindolol, a β-adrenergic receptor antagonist that also has activity as a 5-HT1AR antagonist, to hasten antidepressant response compared with treatment with a SSRI alone.^13, 48, 49, 50^ The investigators of these studies attributed 5-HT1AR activation and decreased 5-HT output as the cause of the delay in SSRI treatment efficacy. Neither pindolol nor any other 5-HT1AR antagonist currently has FDA approval for use in children and adolescents.

Activation of 5-HT1ARs leads to decreased serotonergic neuron firing, and it has been previously hypothesized that this negative-feedback inhibition is the underlying cause of the delay in SSRI efficacy during the initial weeks of treatment.^51, 52^ Furthermore, hyposerotonergic states are associated with increased impulsivity^53^ and SREs.^54^ Therefore, we hypothesized that the rates of loading and clearance of an antidepressant into the body corresponds to the severity of hyposerotonergic states and the incidence of SREs. More specifically, both the loading rate and the clearance rate of an antidepressant dosed once per day should be proportional to the rate of SREs in patients taking that antidepressant. We tested this idea in the present study using *t*_1/2_ data from pediatric and adult SSRI pharmacology studies, and here we demonstrate that the *t*_1/2_ of a SSRI is directly proportional to the relative risk of SREs. We also demonstrate a significant inverse relationship between the time to 90% of *C*_max_ and the rate of SREs, indicating that as time to drug saturation increases, the less likely it is that a patient will experience a SRE. Taken together, these data support the idea that the rates of loading and clearance of an SSRI influence the incidence of negative self-injurious behaviors associated with acute SSRI treatment. Future studies investigating the effect of cognitive and behavioral interventions, such as cognitive behavioral therapy, on 5-HT1A receptor sensitivity to acute SSRI treatment in pediatric patients, might uncover additional means to ameliorate the abrupt induction of hyposerotonergic impulsive states early in the course of antidepressant initiation.

It is important to note that the current analyses utilized the pediatric SRE rates from the 2004 FDA report^20^ and not the 2006 FDA report,^6^ because the 2006 report included the Treatment for Adolescents with Depression Study (TADS). The TADS was the only study in the entire data set of the 2006 report to prospectively measure rates of suicidal thinking and behavior and implement integrated procedures for adverse event monitoring to capture these outcomes. Thus, it should come as no surprise that the risk ratio of fluoxetine-mediated SREs reported in the TADS was vastly different and much higher than the risk ratio of SREs reported from the other four fluoxetine trials measuring SREs retrospectively (4.62 in TADS vs 0.3–1.38 for the other four studies, respectively). It is highly likely that these rates are different due to methodological differences that should preclude its inclusion into an analysis of the other studies. Interestingly, although the TADS study did not report differences between suicidal ideal in children and adolescents treated with fluoxetine versus placebo, fluoxetine treatment did increase the incidence of harm-related events. Therefore, in order to compare SREs between many SSRIs, we only included the retrospectively analyzed studies.

Fluoxetine, the SSRI with the longest half-life, is associated with the lowest number of SREs, as compared with five other SSRIs for which randomized controlled studies are available in children and adolescents. The long half-life of fluoxetine provides consistent central nervous system drug exposure and modest activation of 5-HT1aRs, thereby lowering the risk of hyposerotonergic state induction. We therefore hypothesized that the modification of dosing strategies of other SSRIs to match the daily trough blood concentrations of fluoxetine as closely as possible will decrease the number of SREs associated with the respective SSRIs to the low number reported in patients taking fluoxetine. The calculations conducted in the present study provide the modified dosing regimens that we believe will be both safer and more efficacious than the present inconsistent and unsubstantiated dosing strategies used today. We believe that these escalating dosing proposals might decrease the incidence of deliberate self-harm associated with acute SSRI treatment, as it was recently reported that children and adolescents who begin antidepressant treatment at a high dose have a twofold risk of self-harm behaviors as compared with those who receive modal-dose therapy.^55^ It is also noteworthy that the algorithms used in the present study provide a modified dosing regimen similar to that employed for decades in the treatment of anxiety in adults with SSRIs based solely on empirical clinical observations and experience.^56^ To our knowledge, we are the first to propose a specific dosing strategy, supported by evidence from prior pooled antidepressant studies in children and adolescents, for SSRIs with the purpose of minimizing the risk of SREs in pediatric populations. The enclosed ramped dosing recommendations could sufficiently minimize adverse events associated with other SSRIs and prompt the initiation of clinical trials to approve additional SSRIs (in addition to fluoxetine and escitalopram) for use in children and adolescents. The dosing regimens proposed in the present study were developed for a 70-kg individual, because the available PK parameters for the antidepressants that were included were only available for adults based on calculations employing this average weight. These calculations will be refined when additional PK data become available for SSRIs in pediatric populations, but we are likely to yield the same general dosing strategy assuming that the parameters are employed consistently across all of the drugs analyzed.

Perhaps the adult correlate of the acute negative effects of SSRI treatment is anxiogenesis rather than the impulsivity and aggressiveness engendered by the initiation of SSRI therapy in children and adolescents. The initiation of SSRI treatment in adult populations with anxiety disorders is paradoxically associated with worsening of their anxiousness, despite the anxiolytic effects of this class of medications with chronic administration.^57^ It is probable that increased anxiety and panic attacks in adults that is associated with initiation of SSRI treatment parallels the enhanced SREs seen in children and adolescents, and that these behaviors operate through the same pharmacological mechanisms.

In summary, our data support the hypothesis that decreased anxiety observed in animals treated with a 5-HT1AR antagonist+fluoxetine will translate to both an expedited onset of the beneficial effects of SSRIs along with a decrease in suicidal behaviors in children and adolescents. Co-administration of a 5-HT1AR antagonist with a SSRI could decrease impulsive behavior in newly treated pediatric patients, translating into a decreased risk of suicidal thoughts and behaviors in children and adolescents and decreased anxiety in adults following the initiation of SSRI treatment. Although genetic studies are conflicting regarding the association between 5-HT1AR polymorphisms and impulsivity/suicidal behavior,^58, 59, 60, 61^ the detection of polymorphisms could lead to a personalized pharmacological approach to treating depression in certain affected individuals. In addition, if this combination treatment strategy has a more rapid therapeutic onset, it will be superior to the tapered initial antidepressant dosing regimen in those patients that have time-sensitive treatment needs. Until a selective 5-HT1AR antagonist is approved for use in children, the novel and specific dosing strategies for existing SSRIs that are proposed here provide immediate potential for decreasing the frequency of SREs in pediatric patients with MDD while maximizing the speed of therapeutic effects. Taken as a whole, this work suggests that antidepressants are no different from drugs that are commonly titrated to a therapeutic response (for example, antihypertensive and hypoglycemic medications used to treat hypertension and diabetes, respectively), in that they are not inherently dangerous when their pharmacological properties are understood and dosing regimens are well defined.

## Figures and Tables

**Table 1:** Dosing recommendations for paroxetine, citalopram, sertraline, venlafaxine and fluvoxamine

*Dosing day*	*Fluoxetine* C_*ave*_	*Paroxetine*		*Citalopram*		*Sertraline*		*Venlafaxine*		*Fluvoxamine*	
		*Dose every 24* *h*	C_*ave*_	*Dose every 24 h*	C_*ave*_	*Dose every 24 h*	C_*ave*_	*Dose every 24 h*	C_*ave*_	*Dose every 12 h*	C_*ave*_
1	30.8	19.2	8.7	11.5	10.4	23.7	11.6	82.8	102.4	20.5	15.1
2	55.8	24.6	15.7	13.4	18.8	28.9	20.9	111.2	185.4	21.0	27.3
3	74.8	28.8	21.0	14.7	25.1	33.1	28.0	134.1	248.4	25.0	36.5
4	89.5	31.8	25.2	15.7	30.1	36.4	33.5	152.6	297.2	29.0	43.7
5	101.0	33.6	28.4	16.6	34.0	39.4	37.9	166.5	335.5	31.0	49.3
6	110.1	35.4	31.0	17.4	37.1	41.3	41.3	178.2	365.9	33.0	53.8
7	117.5	36.0	33.0	17.9	39.5	43.1	44.0	187.7	390.3	34.0	57.4
8	123.4	37.8	34.7	18.5	41.5	44.6	46.2	195.3	409.8	36.0	60.3
9	128.1	37.8	36.0	18.8	43.1	45.8	48.0	201.6	425.7	37.0	62.6
10	132.0	39.0	37.1	19.2	44.4	46.9	49.5	206.1	438.5	37.0	64.5
11	135.1	39.0	38.0	19.5	45.5	47.5	50.6	210.6	448.9	38.0	66.0
12	137.7	39.6	38.7	19.6	46.3	48.3	51.6	213.8	457.4	39.0	67.2
13	139.8	39.6	39.3	19.8	47.0	48.8	52.4	216.5	464.3	39.0	68.3
14	141.5	40.2	39.8	20.0	47.6	49.1	53.0	218.7	470.0	40.0	69.1
15	142.9	40.2	40.2	20.2	48.1	49.7	53.6	220.5	474.7	40.0	69.8
16	144.0	40.2	40.5	20.3	48.4	49.8	54.0	222.3	478.5	40.0	70.3
17	145.0	40.8	40.8	20.3	48.8	50.0	54.3	223.2	481.6	41.0	70.8
18	145.7	40.8	41.0	20.4	49.0	50.3	54.6	224.1	484.2	40.0	71.2
19	146.4	40.8	41.2	20.5	49.2	50.6	54.9	225.0	486.3	41.0	71.5
20	146.9	40.8	41.3	20.4	49.4	50.6	55.1	225.9	488.0	41.0	71.7
21	147.3	40.8	41.4	20.6	49.6	50.6	55.2	226.4	489.4	41.0	71.9
22	147.7	40.8	41.5	20.6	49.7	50.7	55.3	226.8	490.6	41.0	72.1
23	148.0	41.4	41.6	20.6	49.8	51.0	55.5	227.3	491.5	41.0	72.3
24	148.2	40.8	41.7	20.7	49.8	50.8	55.5	227.3	492.3	41.0	72.4
25	148.4	40.8	41.7	20.6	49.9	51.0	55.6	227.7	493.0	41.0	72.5
26	148.5	41.4	41.8	20.7	50.0	51.1	55.7	228.2	493.5	41.0	72.6
27	148.7	40.8	41.8	20.6	50.0	50.9	55.7	228.2	494.0	41.0	72.6
28	148.8	41.4	41.9	20.8	50.0	51.2	55.8	228.2	494.3	41.0	72.7
29	148.9	40.8	41.9	20.6	50.1	51.1	55.8	228.6	494.6	41.0	72.7
30	149.0	41.4	41.9	20.7	50.1	51.0	55.8	228.6	494.9	41.0	72.8

If paroxetine, citalopram, sertraline, venlafaxine and fluvoxamine are given based on the following table, their daily mean blood concentrations (*C*_ave_) will be matched to that of fluoxetine at 40 mg every 24 h. Dose is in mg. *C*_ave_ is in ng ml^−1^. Patient's weight is assumed to be 70 kg. *C*_ave_ for venlafaxine is the combination of venlafaxine and its active metabolite, *O*-desmethylvenlafaxine.

## Notes

Drs Rahn, Hendrix and Kaplin report their patent application ‘Methods for reducing anxiety and impulsivity in subjects initiating treatment with serotonin reuptake inhibitors'. The remaining authors declare no conflict of interest.
